# Effect of bronchial thermoplasty on airway inflammatory and profibrotic gene expression after one−year follow−up in severe asthma

**DOI:** 10.3389/fimmu.2026.1886894

**Published:** 2026-08-07

**Authors:** Bogdan Jakiela, Stanislawa Bazan-Socha, Karolina Górka, Iwona Gross-Sondej, Sławomir Mikrut, Krzysztof Okoń, Piotr Sadowski, Anna Andrychiewicz, Tomasz Stachura, Marek Przybyszowski, Grażyna Bochenek, Krzysztof Sładek, Jerzy Soja

**Affiliations:** 1 2nd Department of Internal Medicine, Jagiellonian University Medical College, Kraków, Poland; 2 Department of Pulmonology and Allergology, University Hospital, Kraków, Poland; 3 Faculty of Mining, Surveying and Environmental Engineering, AGH University of Science and Technology, Kraków, Poland; 4 Department of Pathology, Jagiellonian University Medical College, Kraków, Poland; 5 Department of Endoscopy, University Hospital, Kraków, Poland

**Keywords:** airway remodeling, bronchial thermoplasty, gene profile analysis, immunological endotypes, severe asthma

## Abstract

**Background:**

Bronchial thermoplasty (BT) reduces symptoms in severe asthma, but its mechanisms remain poorly understood. This preliminary study aimed to investigate the effect of BT on bronchial gene expression and its associations with airway structural changes.

**Methods:**

Bronchoscopy with endobronchial ultrasound (EBUS), bronchoalveolar lavage (BAL), and endobronchial biopsies was performed in 10 patients with severe asthma before and 12 months after BT. We assessed BAL cytology, biopsy histomorphometry, and mRNA expression of genes related to immune (e.g., innate or type-2 related) and profibrotic responses.

**Results:**

BT resulted in significant improvement in asthma control (ACT score increased from median 14 to 18, *p* = 0.0078) and a reduction in exacerbation rate (from 5 to 1, *p* = 0.0020). Spirometric measures of airflow obstruction did not improve, but air trapping decreased (RV/TLC from 0.47 to 0.39, *p* = 0.0078) after BT. An increase in BAL eosinophils was observed (from 0.3% to 3.7%, *p* = 0.0020), predominantly in patients with greater reductions in systemic corticosteroids. Histological analysis showed a significant reduction in the airway smooth muscle (ASM) mass, while gene-expression data demonstrated only moderate upregulation of remodeling-related transcripts (e.g., *COL3A1*, *GDNF*). These molecular changes were not associated with bronchial structural or spirometric parameters and likely reflect shifts in local inflammatory patterns.

**Conclusion:**

BT does not appear to fundamentally alter the immunological profile in the treated airways but is associated with a decrease in ASM thickness along with features of fibrosis. This was associated with increased BALF eosinophilia, suggesting heterogeneous effect of BT across airway compartments, further modified by corticosteroid reduction accompanying clinical improvement.

## Introduction

Asthma is a chronic respiratory disease with a complex pathogenesis and multiple inflammatory endotypes (1). Structural changes of the airway, collectively referred to as *airway remodeling*, are also characteristic of the disease and usually become more pronounced with increasing asthma severity (2). In bronchial biopsy specimens, airway remodeling is characterized by smooth muscle hyperplasia and hypertrophy, alterations in the composition of extracellular matrix (ECM), goblet cell hyperplasia, and increased vascularity (1, 2). Mechanistically, these changes are linked to various cellular and molecular processes, including epithelial-to-mesenchymal transition and the deposition of collagens and other ECM proteins, ultimately contributing to tissue fibrosis. These mechanisms of airway structural changes are currently the focus of intensive research, particularly in severe asthma. Furthermore, emerging evidence also suggests that novel biologic therapies may exert disease−modifying effects by influencing key processes involved in airway remodeling (2–5).

Bronchial thermoplasty (BT) is a device−based therapy that delivers thermal energy to the airways to target the layer of airway smooth muscle (ASM) cells, reducing their mass and potentially modifying their contractility and other functions (5, 6). Several randomized and observational studies demonstrated clinical benefits of BT, including improvements in quality of life and symptom control in patients with severe asthma, as well as a reduction in exacerbation rates and emergency department visits (6–9). BT results in the reduction in ASM mass in the treated airway, a structural alteration consistently reported in histological evaluations (6, 10, 11). Favorable changes in airway imaging morphometry have also been documented by computed tomography (CT) (12) and endobronchial ultrasound (7, 11, 13). However, these alterations were not associated with improvements in spirometry values (6, 10, 11, 14, 15). Thus, the mechanisms underlying the clinical benefits of BT remain unclear and require further investigation (16, 17).

Only a few studies have analyzed bronchial gene expression profiles before and after BT to clarify the immune and molecular pathways underlying its clinical effects. For example, Facciolongo et al. (18) reported upregulation of genes related to airway remodeling two months after the first BT session. However, it remained unclear whether these changes were stable over time or how the gene expression pattern might evolve during subsequent follow-up. In contrast, Wijsman et al. (6) reported no significant changes in bronchial epithelial gene expression, inflammatory cell profiles, or cytokine levels in bronchoalveolar lavage six months after the BT. Thus, long-term studies are needed to determine whether this procedure induces delayed changes in airway inflammatory response.

Based on this background, we conducted an observational study analyzing the impact of BT on clinical outcomes, airway structural changes, and inflammatory profiles in bronchial mucosa. We compared the expression of genes related to inflammation and remodeling in endobronchial biopsies obtained from the same bronchi before and 12 months after treatment. Our study specifically focuses on identifying associations between local inflammatory changes and structural alterations related to BT.

## Methods

### Patients and study protocol

**Figure 1:**
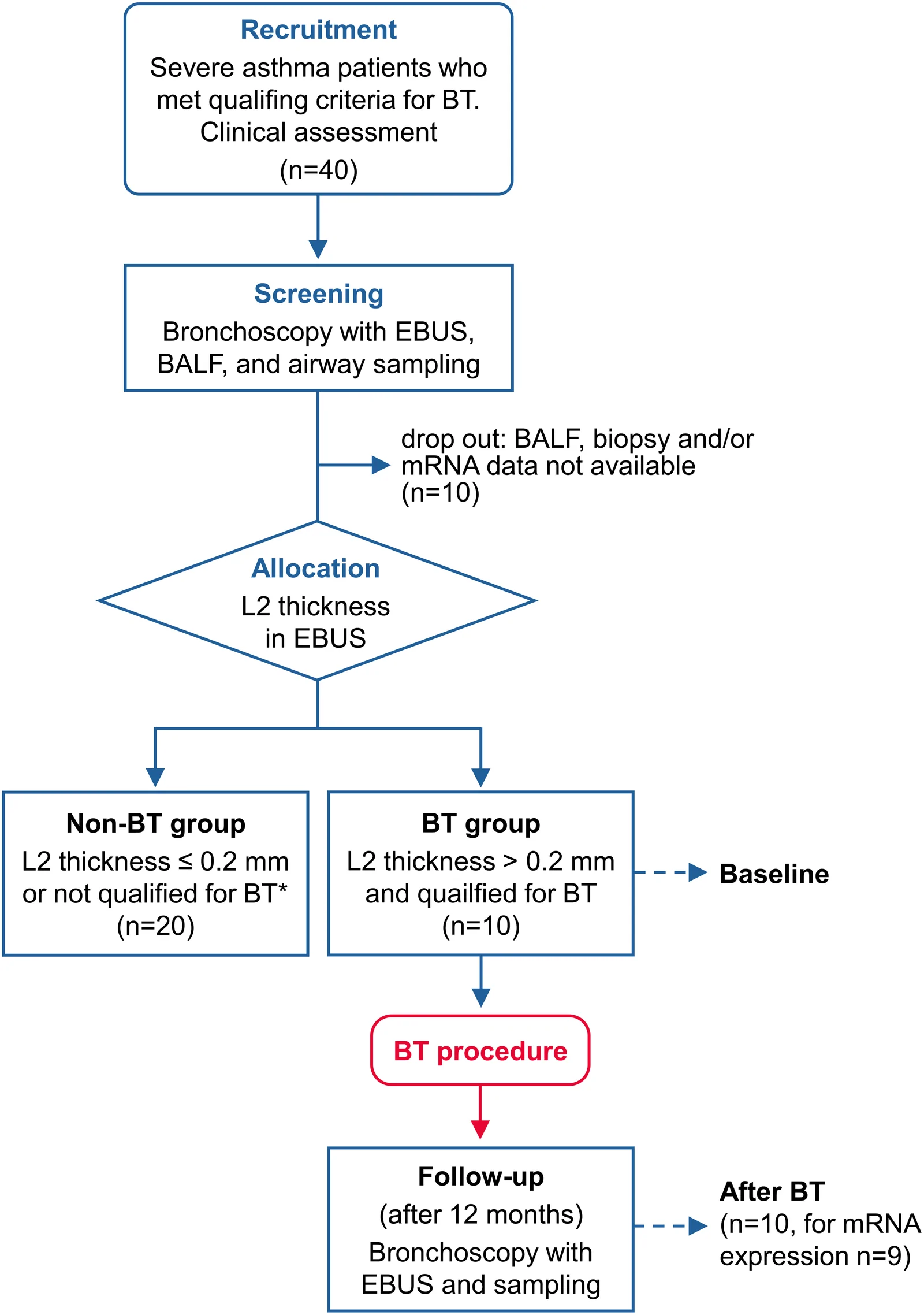
Design of the study. The patients were enrolled as part of a larger project investigating the effectiveness of bronchial thermoplasty (BT) in severe asthma [for ref. see (11)]. In this analysis, we focused on a subset of patients (n=30) for whom complete data on the immunological status of the lower airways were available. Due to the limited availability of the procedure, candidates for BT were selected based on the average L2 thickness measured by EBUS. Ultimately, 10 patients underwent BT and were reassessed at 12-month follow-up. A baseline comparison between the BT and non-BT groups (n=20) is also provided. *Five non-BT patients showed L2 thickness > 0.2 mm but were not eligible for BT due to medical contraindications or declined the procedure.

This prospective observational study enrolled 40 patients with severe asthma at the University Hospital in Krakow, Poland, between 2016 and 2019. All patients fulfilled the criteria for severe asthma according to the Global Initiative for Asthma report (1). None of the participants had received biological therapies in the past or during enrollment. The outline of the study is presented in **Figure 1**. All patients underwent a clinical workup as a part of qualification for BT therapy, which included spirometry with reversibility to a short-acting bronchodilator and measurement of static lung volumes (Jaeger Master Screen, Höchberg, Germany), the Asthma Control Test (ACT) (19), and the Asthma Quality of Life Questionnaire (AQLQ) (20). In all subjects, we performed bronchoscopy with endobronchial ultrasound (EBUS), bronchoalveolar lavage fluid (BALF) collection, and forceps endobronchial biopsy sampling for morphometry and gene expression analysis. Ten patients were excluded from further analysis due to incomplete data (e.g., poor-quality sampled material or other technical issues). In the remaining group (n=30), EBUS measurements were used to identify patients with the thickest L2 layer (> median value of 0.2 mm), who were considered candidates for the BT procedure. Patients undergoing BT were thus selected using an exploratory, study-specific strategy based on EBUS measurements. Ultimately, BT was performed in ten patients, who were evaluated at baseline and at the 12-month follow-up (‘after BT’ in the graphs). After the final BT session, patients remained on their baseline OCS for three months, after which the dose was tapered down according to symptoms and clinical stability. This preliminary study investigates lower airway inflammatory profiles, integrating laboratory and airway structural data, and represents a follow-up to our earlier project focused on the clinical effects of BT (11). Here, we analyze 10 patients (compared to 11 in the previous study) because biopsy gene expression data were not available for one patient who underwent BT. The study protocol followed the Declaration of Helsinki and its amendments and was approved by the Ethics Committee of Jagiellonian University Medical College (KBET 122.6120.167.2015). All participants provided informed written consent.

### Bronchoscopy with EBUS and airway sample workup

Bronchoscopy with EBUS and bronchial sampling was performed under local anesthesia and conscious sedation using the BF-190 fiberscope (Olympus, Tokyo, Japan) as previously described (11). In brief, EBUS was conducted in the four segmental bronchi of the right lower lobe (RB6, RB8, RB9, and RB10) using a 20 MHz radial probe (Olympus). The details of EBUS methodology and data analysis workflow are provided in **Supplementary Figure 1**. The acquired images were analyzed using feature-extraction software to measure the average thickness of the bronchial wall layers: L1 (epithelium and the proximal submucosa), L2 (the majority of submucosa, including smooth muscle), and combined L3-L5 (cartilage). BALF was collected from the segmental bronchus of the right middle lobe after instilling 0.9% saline solution (four aliquots, 200 mL total). BALF cell differential counts were determined using cytospin preparations (Thermo Fisher Scientific, Waltham, MA). Endobronchial biopsies were obtained from the segmental bronchi carinae of the right lower lobe (RB6, RB8, RB9 and RB10) at comparable locations before and after BT, and thus correspond to treated bronchial regions. A total of six biopsies per procedure were collected using conventional biopsy forceps (EndoJaw; Olympus). Tissue samples were either fixed in 10% buffered formalin for histological analysis or mixed with TRI Reagent (Sigma-Aldrich, St. Louis, MO) and stored at -80 °C for RNA extraction.

### Bronchial thermoplasty

BT was performed using the Alair™ Bronchial Thermoplasty System (Boston Scientific, Natick, MA, USA) under general anesthesia (propofol) and using the laryngeal mask airway. A single-use catheter equipped with a basket of four expandable electrodes was introduced into the bronchial tree to deliver radiofrequency thermal energy to the airway wall. Ablations were applied to short, approximately 5-millimeter segments in bronchi measuring 3–10 mm in diameter. BT was carried out in accordance with practice guidelines (21), in three separate sessions with 3-week intervals, consecutively for the right lower lobe, the left lower lobe, and, finally, both upper lobes. A prednisone course (50 mg/d) was administered for 3 days before BT, and continued until one day after the procedure.

### Bronchial biopsy histology

Formalin-fixed endobronchial biopsy samples were routinely processed, sectioned at 3 µm, and stained with hematoxylin-eosin or immunohistochemically with antibody detecting alpha-smooth muscle actin (α-SMA; M0851; Dako Denmark A/S, Glostrup, Denmark) or collagen-I (polyclonal; Abcam, Cambridge, United Kingdom). The specimens were analyzed using an Olympus SC 180 camera (Olympus) and the Olympus cellSens Standard 2.3 software. ASM content was expressed as the ratio of α-SMA-positive area to the area of the entire section. Reticular basement membrane (RBM) thickness was calculated as the mean of at least 30 individual measurements obtained along the available epithelium length using the AnalySIS imaging system (Soft Imaging System GmbH; Germany) and a custom-built application. The intensity of collagen staining was scored 0–3 and averaged across various areas of the specimen. All histological examinations were performed by experienced pathologists who were blinded to the clinical data.

### RNA isolation and real-time PCR

Total RNA was isolated from endobronchial biopsies using the Trizol method (Sigma-Aldrich, St. Louis, MO) with silica-spin columns (A&A Biotechnology, Gdynia, Poland) and subsequently reverse transcribed (High-Capacity cDNA Reverse Transcription Kit; Applied Biosystems, Foster City, CA). Relative mRNA expression was quantified using TaqMan low-density arrays (assays listed in **Supplementary Table 1**) on the QuantStudio 12K Flex Real-Time PCR System (Applied Biosystems) as previously described (8). RNA was extracted from whole endobronchial biopsy specimens; therefore, gene expression results represent a composite signal from multiple compartments of the bronchial mucosa. qPCR data were normalized to the reference gene *GAPDH*, and relative expression differences at 12-month follow-up compared to baseline were calculated using a 2-ΔΔCT method. The results were controlled for multiple comparisons using the Benjamini-Hochberg correction at the false discovery rate (FDR) threshold *q* = 0.1. Thresholds for T2 and T3 asthma signatures were based on our earlier analysis of the entire severe asthma cohort (8).

### Statistics

Data were analyzed using Statistica 13.1 (TIBCO Software Inc., Palo Alto, CA) and GraphPad Prism 8.4 (GraphPad Software, Inc., La Jolla, CA). In Tables and graphs, data are presented as medians with quartiles (Q1-Q3) or as counts with percentages. Group comparisons were performed using Mann-Whitney (non-BT vs BT groups) or Wilcoxon tests (effect of BT) for continuous data, and Fisher’s exact test for categorical variables. Correlations were presented as heat maps of Spearman (r_S_) coefficients, with examples shown in accompanying scatter plots. A *p*-value < 0.05 was considered statistically significant. Given the small cohort in the BT arm, resulting from the limited availability of the procedure, no formal sample size calculation was performed and analyses should be considered exploratory, with emphasis on effect sizes and consistency of responses. For clarity, the number of replicates, statistical tests, and the *p*-value thresholds are specified in each figure and table legend.

## Results

### Bronchial thermoplasty is associated with significant clinical improvement in severe asthma

Of the 40 severe asthma patients recruited to the study, 10 with increased L2 layer thickness on EBUS were eligible for the BT procedure. The BT group consisted of 4 women and 6 men, with a median age of 50.5 years, a body mass index of 25.4 kg/m^2^, and an asthma duration of 15.5 years (**Supplementary Table 2**). Most patients had adult-onset asthma (n=8, 80%), and all were non-smokers. Patients who qualified for BT showed clinical characteristics very similar to the remaining severe asthmatics (non-BT), except for a higher daily dose of inhaled corticosteroids (median 2200 vs. 1450 µg/d, *p* = 0.0151). Although the BT group exhibited increased airway wall thickness across all EBUS layers, lung function measures were comparable to those of non-BT subjects. Full baseline characteristics are provided in **Supplementary Table 2**.

All 10 eligible patients completed three BT sessions without serious adverse events. Patients were subsequently monitored, and bronchoscopy with EBUS and airway sampling was repeated after 12 months of follow-up (**Figure 1**). As we reported previously (11), BT resulted in significant improvement in asthma control. In the present analysis, we observed a 28.6% increase in ACT score, an 80.0% decrease in asthma exacerbation rate, a 60.0% reduction in oral steroid use, and a 27.27% reduction in daily inhaled steroid dose (**Table 1**). Consistent with earlier studies (6, 10, 11, 14, 15), spirometric measures of airway obstruction did not improve significantly after BT. For example, FEV_1_ (% predicted) increased from a median of 63.5% at baseline to 70.1% after BT (*p* = 0.194). However, at 12-month follow-up, patients showed reduced FEV_1_ reversibility after bronchodilator, decreasing from 8% to 3% (*p* = 0.0488). Notably, BT led to a marked reduction in air trapping, reflected by 14.8% decrease in total lung capacity (TLC), a 31.2% decrease in residual volume (RV), and a 17.0% reduction in the RV/TLC ratio (*p* = 0.0078) (**Table 2**). Furthermore, as previously described (11), BT resulted in a significant reduction in airway wall thickness on EBUS and in the ASM area on biopsy morphometry (**Table 2**).

**Table 1:** Clinical characteristics and BALF cell differential at baseline and after BT.

n=10	Baseline	After BT	Median difference (95% CI)	*p*-value
ACT (score)	14 [11–19]	18 [15–24]	3.5 (0.7, 8.5)	**0.008**
AQLQ (score)	4.38 [3.22-5.71]	5.86 [4.37-6.13]	0.57 (-0.14, 1.83)	0.055
Exacerbations (n/year)	5 [3-8]	1 [1-3]	-4 (-5, -2)	**0.002**
ICS (µg/d)^1^	2200 [1950-2425]	1600 [735-2425]	-500 (-1195, -30)	**0.035**
OCS (mg/d)^2^	10 [0-16]	4 [0-10]	-2 (-7, 1.3)	0.219
OCS (n, %)	7 (70%)	6 (60%)	n.a.	>0.999
Blood eosinophils (cells/µL)	156 [71-238]	110 [30-268]	-44 (-184, 165)	0.625
Blood basophils (cells/µL)	18 [9-25]	24 [17-61]	9 (-5, 33)	0.201
Blood neutrophils (cells/µL)	4026 [2908-5762]	4221 [2660-6747]	665 (-1736, 2502)	0.625
Serum total IgE (IU/mL)	70.9 [58.1-295]	78.5 [42.2-328]	1.6 (-116, 73.4)	0.938
Serum CRP (mg/L)	1.7 [1.0-8.8]	1.0 [1.0-2.1]	0.0 (-20, 8)	0.672
BALF analysis				
BALF cell count (10^3^ cells/mL)	126 [91-169]	138 [113-153]	24 (-25, 42)	0.539
Macrophages (% of non-epi. cells)	92.4 [89.3-96.6]	84.6 [74.2-90.9]	-4.8 (-19.3, 1.3)	0.084
Neutrophils (% of non-epi. cells)	1.23 [0.48-2.18]	1.06 [0.28-4.70]	-0.01 (-2.26, 5.95)	0.922
Eosinophils (% of non-epi. cells)	0.34 [0.09-1.56]	3.74 [0.39-15.8]	3.39 (0.06, 17.2)	**0.002**
Lymphocytes (% of non-epi. cells)	5.15 [2.39-8.47]	4.20 [3.20-5.78]	-0.73 (-7.50, 4.41)	0.492
BALF Eosinophils >2% (n, %)	1 (10%)	6 (60%)	n.a.	0.057
BALF Neutrophils >3% (n, %)	1 (10%)	2 (20%)	n.a.	>0.999
Airway inflammation, E/Mix/N/P^3^	1/0/1/8	5/1/1/3	n.a.	0.086

ACT, Asthma Control Test; AQLQ, Asthma Quality of Life Questionnaire; BALF, bronchoalveolar lavage fluid; BMI, body mass index; BT, bronchial thermoplasty; CRP, C-reactive protein; ICS, inhaled corticosteroids (fluticasone-equivalent); n.a., not applicable; non-epi, non-epithelial; OCS, oral corticosteroids (methylprednisolone-equivalent).

References: 1, dose adjusted for fluticasone propionate; 2, dose adjusted for methylprednisolone; 3, incidence of lower airway inflammatory phenotypes in asthma patients based of BALF cell analysis: E, eosinophilic (>2% eosinophils, ≤3% neutrophils); N, neutrophilic (≤2% eosinophils, >3% neutrophils); Mix, mixed (both increased); P, pauci-granulocytic (≤2% eosinophils, ≤3% neutrophils).

Statistics: Data are presented as medians [Q1-Q3 quartiles]. Median differences with Wilcoxon statistics (for continuous data) or contingency table statistics (for categorical data; Fisher’s exact test, Chi-square for E/N/P distribution) are presented in separate columns. Significant differences (*p* < 0.05) are highlighted with a bold font.

**Table 2:** Lung function, EBUS measurements, and endobronchial biopsy histology at baseline and after BT.

n=10	Baseline	After BT	Median difference (95% CI)	*p*-value
Lung function				
FVC (% predicted)	78.4 [66.6-90.0]	84.8 [76.6-89.1]	1.7 (-2.7, 11.9)	0.232
FEV1 (% predicted)	63.5 [47.0-71.3]	70.1 [52.6-74.2]	2.5 (-1.6, 10.0)	0.193
FEV1%FVC before BD	62.6 [54.8-68.7]	65.7 [60.6-69.5]	4.1 (-0.5, 6.8)	0.084
FEV1%FVC after BD	65.6 [60.0-75.3]	66.9 [59.3-79.4]	1.3 (-2.2, 3.4)	0.432
FAO (n,%)	6 (60%)	6 (60%)	n.a.	>0.999
FEV1.rev (% change after BD)	8.0 [2.4-19.7]	3.0 [1.1-9.3]	-2.0 (-10.2, -0.01)	**0.045**
TLC (% predicted)	118 [94.5-123]	100.5 [87.5-109]	-8.7 (-15.0, -2.9)	**0.020**
RV (% predicted)	177 [106.5-209]	121.7 [94.8-148]	-31 (-59, -15)	**0.004**
RV/TLC (ratio)	0.47 [0.34-0.52]	0.39 [0.35-0.47]	-0.05 (-0.11, -0.02)	**0.008**
EBUS measurements				
L1 (mm)	0.185 [0.179-0.191]	0.173 [0.168-0.176]	-0.011 (-0.016, -0.008)	**0.002**
L2 (mm)	0.208 [0.202-0.213]	0.185 [0.181-0.194]	-0.020 (-0.027, -0.015)	**0.002**
L3-5 (mm)	0.979 [0.953-1.019]	0.885 [0.853-0.890]	-0.104 (-0.146, -0.071)	**0.002**
Bronchial biopsy histology				
ASM (% of biopsy area)	10.3 [5.5-14.6]	4.0 [1.3-6.3]	-6.1 (-11.1, -2.6)	**0.004**
RBM thickness (µm)	6.96 [6.20-8.58]	6.65 [5.48-7.40]	-1.02 (-1.91, 0.16)	0.084
Collagen (staining score)	0.88 [0.75-1.38]	1.25 [0.75-1.68]	0.29 (-0.26, 0.59)	0.375

ASM, airway smooth muscle; BD, bronchodilator (short acting β2-agonist); BT, bronchial thermoplasty; FAO, fixed airway obstruction (defined as FEV_1_/FVC post BD <70%); FEV1, forced expiratory volume in 1 second; FEV1.rev, reversibility of FEV1; FVC, forced vital capacity; n.a., non applicable; RBM, reticular basement membrane; RV, residual volume; TLC, total lung capacity. EBUS data are expressed as the average thickness of bronchial wall layers: L1 (epithelium and the proximal submucosa), L2 (the majority of the submucosa, including smooth muscle), and combined outer layers L3-L5 (representing cartilage).

Statistics: Data are presented as medians [Q1-Q3 quartiles]. Median differences with Wilcoxon statistics (for continuous data) or contingency table statistics (for FAO; Fisher’s exact test) are presented in separate columns. Significant differences (*p* < 0.05) are highlighted with a bold font.

### Minor changes in the airway epithelium expression profile of immune genes after BT suggest the stability of inflammatory endotypes in severe asthma

**Figure 2:**
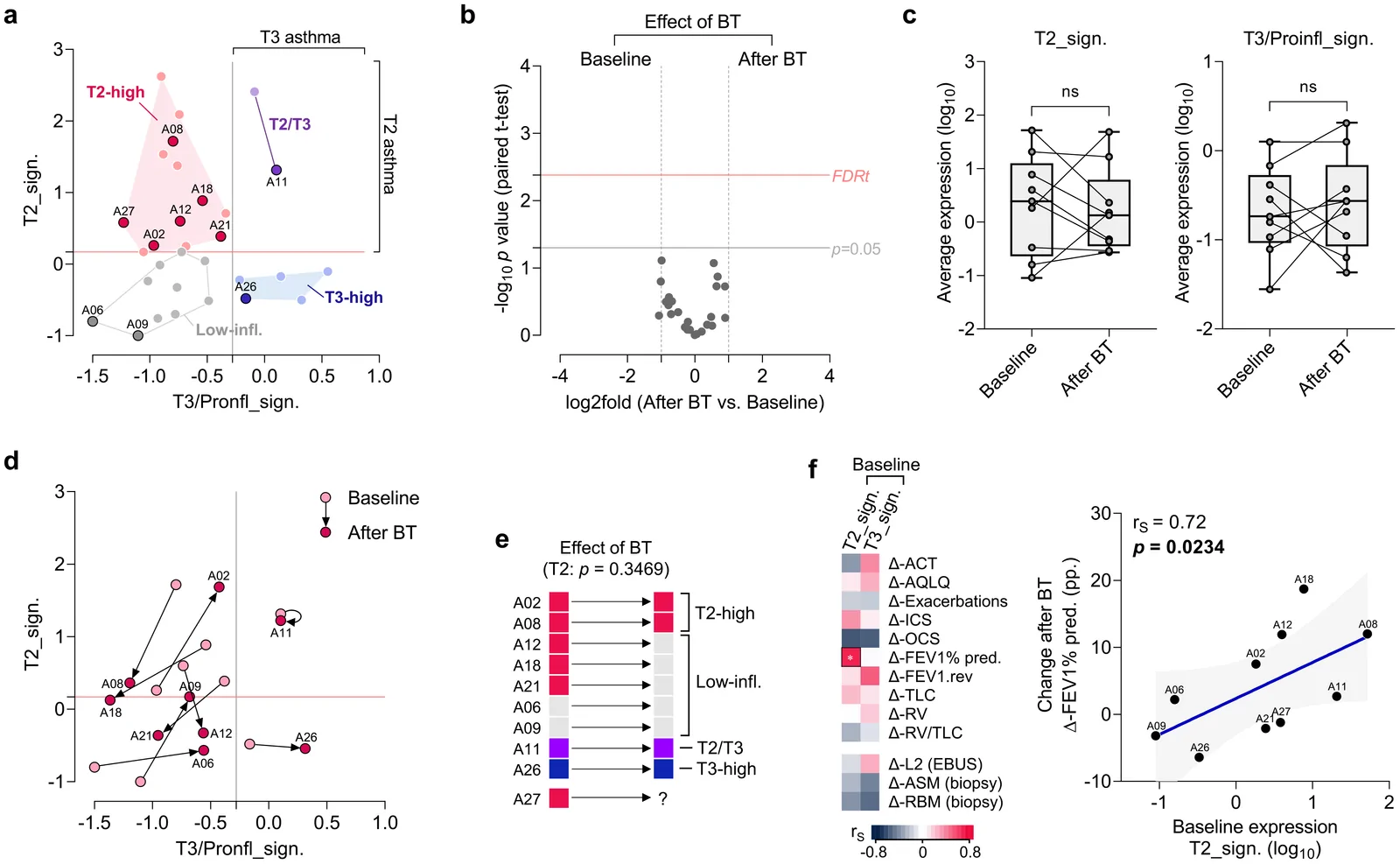
Minor changes in bronchial biopsy expression of immune signature genes after BT. **(A)** Average expression of T2 and T3/proinflammatory genes in endobronchial biopsies sampled at baseline in severe asthma patients included in the study (n=30). Cutoffs for T2 and T3 gene expression patterns and the resulting inflammatory endotypes of severe asthma were determined as previously described (for ref. see (8)). Numbers indicate patients who underwent the BT (n=9). **(B)** Similar mRNA expression of immune genes in endobronchial biopsies obtained one year after BT compared with baseline (n=9). 2-sided paired t-test, FDRt *q* = 0.1. Vertical dashed lines indicate a 2-fold difference. **(C)** No difference in the average expression of T2 and T3/proinflammatory genes before and after BT. Data shown as medians with quartiles (n=9). Wilcoxon test; ns, non-significant. **(D)** BT-related changes in expression of immune signature genes are plotted against the baseline gene expression pattern obtained in the entire severe asthma cohort (as in ‘a’). Arrows indicate changes one year after BT. **(E)** Effect of BT on the prevalence of immune endotypes (Fisher’s test; post-BT data not available for patient A27). **(F)** Correlation matrix (rS, Spearman coefficients) between baseline T2 or T3/proinflammatory signature and post-BT change (Δ, delta) in key clinical and structural measures (n=9; *p < 0.05). The example in the right panel shows the correlation between the T2 mRNA signature and FEV_1_% (fitted regression line with 95% CI). ACT, asthma control test; AQLQ, asthma quality of life questionnaire; ICS, inhaled corticosteroids (fluticasone-equivalent); OCS, oral corticosteroids (methylprednisolone-equivalent); FEV_1_, forced expiratory volume in 1 sec.; TLC, total lung capacity; RV, residual volume; ASM, airway smooth muscle mass; RBM, reticular basement membrane thickness. .

The primary aim of this study was to investigate how BT influenced immune signatures in bronchial biopsies. By analyzing the baseline expression of key T2 (e.g., *CST1*) and T3/proinflammatory (e.g., *IL17A*) genes described in our earlier work (8), we first identified major immune endotypes in the entire severe asthma cohort (**Figure 2A**). Most patients who qualified for BT exhibited a sole T2-high signature (n=6, 60%), while the remaining patients showed T3-high (n=1), mixed (n=2), or low-inflammatory profiles (n=2). Analysis of biopsies collected one year after BT revealed no substantial changes in the expression of immune genes, compared to baseline (**Figure 2B**). Similarly, the average expression of T2 and T3 signature genes did not differ significantly (**Figure 2C**). Tracking individual trajectories (**Figure 2D**) showed mainly variable changes in T2-related gene expression, whereas the T3/proinflammatory signature remained relatively stable. Notably, only three T2-high asthmatics transitioned to a low-inflammatory endotype after BT, while the remaining patients retained their baseline immune profile (**Figure 2E**). The distribution of immune endotypes before and after BT did not differ significantly (e.g., T2 vs. other: Chi^2^, *p* = 0.3469).

Next, we examined whether the baseline immune profile influenced BT outcome. We did not detect any clear association between baseline bronchial biopsy signatures and the overall effect of BT (**Figure 2F**), except for a positive correlation between T2 mRNA signature and post-BT improvement in FEV_1_% (r_S_ = 0.72, *p* = 0.0234). Additionally, we observed a weak negative association between the baseline T2 signature and the reduction in oral corticosteroid dose (r_S_ = –0.59, *p* = 0.0789). These findings suggest that the BT was associated with greater improvement in airway obstruction and a more pronounced oral corticosteroid-sparing effect in patients with T2-high asthma. Notably, changes in airway structural measures did not correlate with baseline immune signatures (e.g., baseline T2_sign. vs. delta-ASM: r_S_ = –0.25, *p* = 0.4918; T2_sign. vs. delta-L2: r_S_ = –0.12, *p* = 0.7589), indicating that BT-related reduction in ASM mass and airway wall thickness was independent of asthma immune endotype. However, the small sample size and the predominance of T2-high patients limited our ability to perform a more robust comparison of BT effects in T2 versus non-T2 asthma.

### BT is associated with an increased number of inflammatory cells in BALF

**Figure 3:**
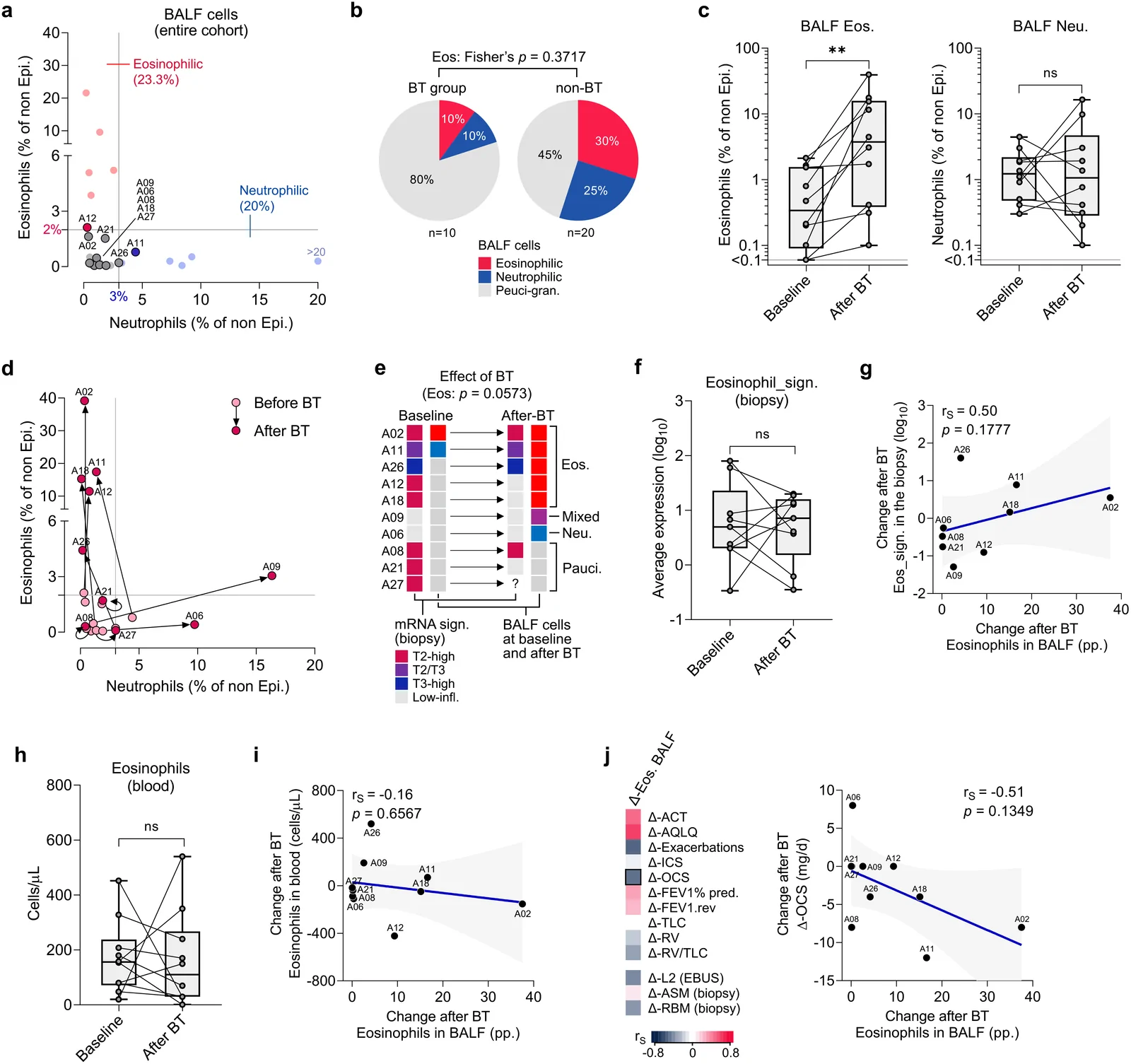
Divergent changes in BALF inflammatory cell profiles one year after BT, with a frequent rise in eosinophils. **(A)** Percentage of bronchoalveolar lavage fluid (BALF) neutrophils and eosinophils in the entire asthma cohort studied (n=30). Labels indicate patients who underwent the BT (n=10). **(B)** Distribution of inflammatory phenotypes in patients qualified for BT (BT group) compared to the remaining patients (non-BT). **(C)** Changes in the percentage of BALF eosinophils and neutrophils before and after BT. Data are shown as medians with quartiles (n=10). Wilcoxon test; **p < 0.01; ns, non significant. **(D)** Individual changes in the percentage of BALF granulocytes and **(E)** corresponding shifts in inflammatory phenotypes before and after BT. Fisher’s exact test results are shown above the graph. **(F)** Average mRNA expression of eosinophil signature genes (*CLC*, *CCR3*, and *PRSS33*) in bronchial biopsies before and after BT (n=9). **(G)** Lack of correlation between the BT-related change in BALF eosinophils (pp., percentage points) and fold change in eosinophilic signature gene expression in the biopsy (fitted regression line with 95%CI range). r_S_, Spearman coefficient. **(H)** Changes in blood eosinophil counts related to BT procedure and **(I)** lack of correlation with the increase in BALF eosinophils. **(J)** Correlation matrix showing association between BT-related increase in BALF eosinophils (Δ-Eos) and changes in clinical and airway structural measures (n=10). All correlations were non-significant (*p* > 0.05). The example plot on the right illustrates a weak negative correlation between Δ-Eos in BALF and reduction in oral corticosteroids after BT (Δ-OCS). ACT, asthma control test; AQLQ, asthma quality of life questionnaire; ASM, airway smooth muscle mass; BALF, bronchoalveolar lavage fluid; BT, bronchial thermoplasty; EBUS, endobronchial ultrasound; Epi, epithelial cells; Eos, eosinophils; FEV1, forced expiratory volume in 1 sec.; ICS/OCS, inhaled/oral corticosteroids; Neu, neutrophils; RBM, reticular basement membrane thickness; RV, residual volume; TLC, total lung capacity.

In the next part of the study, we examined the effects of BT on inflammatory cells in BALF. Baseline analysis of the entire asthma group revealed a predominantly pauci-granulocytic pattern (n=17, 56.7%), with the remaining patients showing eosinophilic (>2% of BALF eosinophils; n=7, 23.3%) or neutrophilic (>3% neutrophils; n=6, 20%) inflammation (**Figure 3A**). Patients who qualified for BT were characterized by relatively low numbers of BALF granulocytes and were primarily classified as pauci-granulocytic (n=8, 80%), with no significant difference compared to non-BT group (**Figure 3B**). Unexpectedly, one year after BT most patients showed a considerable increase in the percentage of BALF eosinophils (from a median 0.34% at baseline to 3.74% after-BT, Wilcoxon *p* = 0.0050; **Figures 3C**, left). In contrast, BALF neutrophils did not change significantly after BT, with only two patients demonstrating a noticeable increase (**Figures 3C**, right). Analysis of individual BALF profiles (**Figure 3D**) revealed that changes in inflammatory cells followed two distinct trajectories (**Figure 3E**): pauci-to-eosinophilic (patients A02, A11, A12, A18, A26) or pauci-to-neutrophilic (patients A09, A06). The former pattern correlated primarily with the baseline T2 signature, whereas the latter aligned with the low-inflammatory signature. However, no consistent association was observed between the mRNA expression patterns in bronchial biopsy samples and the corresponding changes in BALF (**Figure 3E**). Unexpectedly, average expression of eosinophil signature genes in bronchial biopsies did not increase after BT (**Figure 3F**) and was not associated with changes in BALF eosinophil percentages (**Figure 3G**). Similarly, blood eosinophil counts remained unchanged before and after BT (**Figure 3H**) and showed no correlation with BALF findings (**Figure 3I**). The increase in BALF eosinophils after BT showed weak negative correlation with the reduction in oral corticosteroid dose (r_S_ = -0.51, *p* = 0.1349), but no associations were found with other clinical measures (e.g., reduction in inhaled corticosteroids or number of exacerbations) or with airway remodeling parameters (**Figure 3J**).

### Only moderate changes in the bronchial expression of profibrotic genes after BT

**Figure 4:**
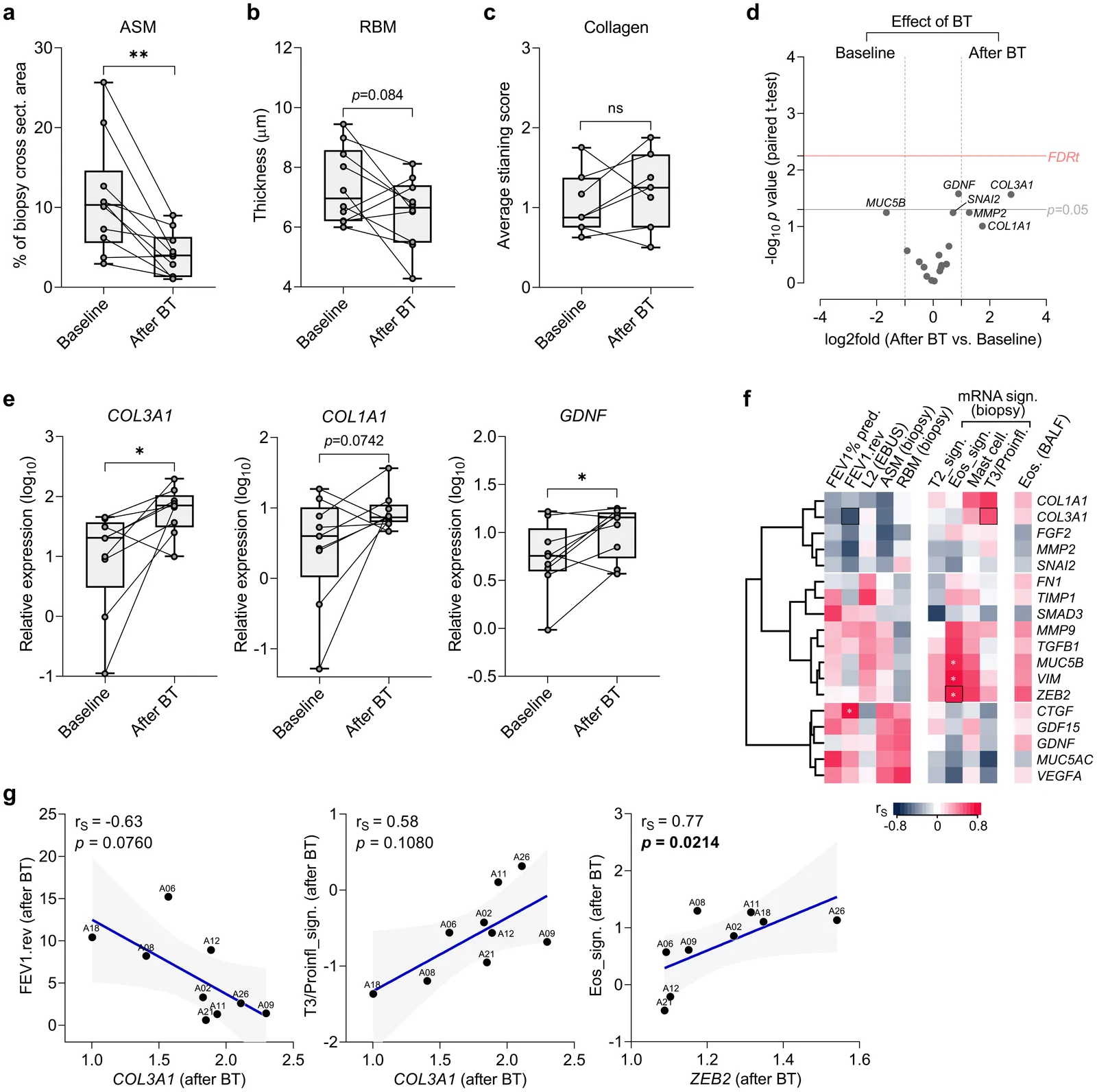
Small changes in bronchial expression of remodeling genes after BT. **(A)** Reduction in airway smooth muscle (ASM) content related to BT. Data expressed as medians and quartiles (n=10). Wilcoxon test; **p < 0.01. **(B)** Changes in reticular basement membrane (RBM) thickness after BT. **(C)** Staining intensity of collagen deposits (n=7). **(D)** Expression difference of remodeling genes in endobronchial biopsies 1 year after BT compared to baseline (n=9). 2-sided paired t-test, FDRt *q* = 0.1. Vertical dashed lines indicate 2-fold difference. **(E)** Bronchial biopsy mRNA expression of *COL3A1*, *COL1A1*, and *GDNF* before and after BT. Wilcoxon test; *p < 0.05. **(F)** Matrix of coefficients (Spearman, r_S_; *p < 0.05.) and cluster classification (Ward, Euclidean dist.) of data collected after BT (n=9) showing correlations between expression of remodeling genes (*side*) and important variables related to lung function, airway structural measures, and bronchial biopsy immune profile (*top*). **(G)** Exemplary correlation plots (indicated with squares in ‘e’ panel matrix) showing association between biopsy expression of *COL3A1* with FEV_1_% reversibility after bronchodilator and expression of T3 and proinflammatory genes, and between biopsy expression of *ZEB2* and eosinophil signature genes (fitted regression lines with 95%CI). For abbreviations, see **Figure 3** legend.

In the last part of the study, we hypothesized that the discrepancy in inflammatory patterns observed in large bronchi compared to BALF could result from bronchial mucosal scarring and more advanced fibrosis in response to the BT procedure. As already described in our earlier study (11), BT resulted in a marked decrease in ASM content in bronchial biopsy specimen (**Figure 4A**; from a median of 10.3 at baseline to 4.0% after BT, *p* = 0.0039) and a slight decrease in the bronchial layer thickness in EBUS (summary in **Table 2**). Measurements of RBM thickness showed a trend toward reduction after BT (**Figure 4B**; from 6.96 to 6.65 µm, *p* = 0.084), whereas collagen staining scores varied largely and did not change significantly (**Figure 4C**; *p* = 0.375). Because biopsies were taken from the same bronchi that underwent BT, these findings indicate that the procedure leads to a substantial reduction in submucosal ASM mass and a modest thinning of the bronchial wall, but is not associated with increased collagen deposition in the mucosa.

Next, we examined whether bronchial mRNA expression of remodeling-related genes corresponded to those histological changes. In the analyzed panel, we included genes encoding key growth factors (e.g., *TGFB1*), transcription factors (e.g., *ZEB2*), and extracellular matrix proteins (e.g., *COL1A1*). In bronchial samples obtained one year after BT we observed increase in expression of *COL3A1* (average 6.7-fold) and *GDNF* (1.9-fold), as well as trends toward increased expression of *COL1A1*, *MMP2* and *SNAI2* (**Figure 4D**). Expression of collagen genes in particular showed concordant BT-related changes (**Figure 4E**). However, these differences did not hold significance after correction for multiple comparisons (FDRt threshold in **Figure 4D**). We also identified notable associations between post-BT expression of remodeling genes and airway structural and immune-related variables (**Figure 4F**). In particular, expression of collagen genes showed weak negative correlation with FEV_1_ reversibility at 12-month follow-up (e.g., for *COL3A1*, Spearman r_S_ = –0.63, *p* = 0.076) and ASM mass (r_S_ = –0.52, *p* = 0.1618), and a positive correlation with T3/proinflammatory signature in the biopsy (**Figure 4G**). Conversely, a subset of remodeling genes, including *TGFB1* and *ZEB2*, positively correlated with eosinophil and mast cells signature markers associated with T2 pattern (e.g., *ZEB2*, r_S_ = 0.77 for Eos_sign, **Figures 4G, H**). BALF eosinophilia showed much weaker associations with bronchial remodeling markers (e.g., *ZEB2*, r_S_ = 0.50 for BALF eosinophils). Of note, bronchial biopsy morphometry measures did not show uniform relationship with remodeling transcripts (**Figure 4F**). For example, expression of most growth factor genes tended to correlate positively with ASM mass and RBM thickness (e.g., *VEGFA*, r_S_ = 0.67 for RBM), whereas collagens genes showed the opposite trend (e.g., *COL1A1*, r_S_ = –0.53 for ASM). Additionally, BT-related changes in expression of collagen genes showed weak association with improvements in ventilatory parameters (RV/TLC and FEV1 reversibility) as well as with reduction in L2 thickness (**Supplementary Figure 2**). Given the exploratory nature of the study and the limited sample size, molecular findings showing only trend-level significance after correction for multiple comparisons should be interpreted with caution.

Overall, these findings indicate that BT leaves a distinct molecular imprint on treated bronchi, reflected by modest increases in remodeling gene expression (e.g., collagens). However, the broader remodeling gene expression profile appears to be shaped predominantly by the prevailing type of airway inflammation, with unique contributions from both T3 and T2 immune signatures.

## Discussion

In this preliminary study, we investigated the effects of BT on clinical outcomes, airway structural measures, and airway inflammatory profiles in severe asthma patients with advanced airway remodeling. BT resulted in substantial clinical improvement, accompanied by decrease in airway wall thickness and reduced ASM mass, yet it did not lead to marked alterations in expression of immune or remodeling-related genes in the bronchial mucosa. Biopsies obtained from the treated bronchi one year after BT showed only a modest increase in expression of selected profibrotic genes, including those encoding fibrillar collagens, *GDNF* (glial cell line-derived neurotrophic), and *SNAI2*, which encodes a transcription factor involved in epithelial-mesenchymal transition (22). However, gene expression patterns reflecting inflammatory endotypes of asthma did not change substantially. Unexpectedly, most patients showed an increase in BALF eosinophils one year after BT, a finding likely related to the reduction in oral corticosteroid doses.

BT is a relatively new but effective therapeutic option for patients with asthma who do not respond adequately to conventional treatment (6, 7, 9). Clinical benefits of BT appear to be sustained, with some studies reporting improvement for up to 10 years of follow-up (16). However, the exact mechanism of BT effects and the asthma phenotypes most likely to benefit from the procedure remain unclear (1, 7). Although the range of biological therapies in asthma continues to expand, clinical remission in severe disease is achieved in only about 30% of patients (23). Moreover, a proportion of patients are not eligible for biologics, do not tolerate them well, or experience significant adverse events (24). In such situations, BT may represent a valuable alternative, particularly for patients with T2-mediated inflammation who remain unresponsive to biologics. Supporting this concept, Angsuwatcharakorn et al. (25) recently demonstrated that BT was indeed clinically effective in a small group of patients with prior biologic failure. In our cohort, individuals with T2-high asthma also responded well to BT, at least in terms of oral corticosteroid sparing and improved FEV_1_ reversibility. Additionally, some experts have proposed that BT may be considered in selected cases of difficult-to-control asthma, including those related to nonadherence to medications, poor inhaler technique, comorbidities, or behavioral factors (7).

BT delivers radiofrequency energy to a limited number of central medium- to large-sized airways, yet its precise mechanism of action remains a matter of debate. Proposed explanations include effects on the airway epithelium, submucosal glands, and airway innervation, each of which may trigger secondary changes in the small airways. The ASM layer is a key contributor to structural remodeling in severe asthma, and is thus widely considered as the primary target for BT (26). Consistent with this concept, we observed a reduction in ASM mass in histological assessment. Similar findings were reported, e.g., in the ASMATHERM study, which also demonstrated decreases in the number of neuroendocrine epithelial cells and bronchial nerve endings, without accompanying changes in mucous glands or ECM accumulation (27). Although we did not assess epithelial damage, Chernyavsky et al. (26) reported improvements in epithelial integrity and suggested that epithelial repair might contribute to the clinical benefits of BT. This finding aligns with the TASMA study, which showed an expanded epithelial basal cell population in bronchial brushes from BT-treated airways (10, 28), likely reflecting epithelial regeneration. BT may also influence a broader range of pathogenetic mechanisms in asthma, extending beyond the direct thermal effects on the airway wall. For example, experimental studies have shown that BT disrupts pulmonary neuroendocrine cell function and signaling (29), and alters metabolic pathways in epithelial cells (30). Some of these changes may reflect direct physical effects of BT, whereas others may represent tissue repair processes occurring in deeper airway wall layers, such as those associated with ASM hypertrophy. Perhaps this mechanistic heterogeneity partially explains the variability observed in clinical outcomes following BT. Although in our study the procedure markedly reduced exacerbation frequency, improvements in symptom-based outcomes were modest, with a proportion of patients remaining partially uncontrolled. This discrepancy may reflect the presence of fixed airway obstruction and ongoing treatment adjustments, as well as the possibility that BT primarily reduces the susceptibility to exacerbations rather than fully normalizing the burden of daily symptoms. Variability in asthma control outcomes has also been demonstrated in recent meta-analyses of randomized controlled trials evaluating BT (31).

In our study, the bronchial immune gene expression profile did not change significantly, consistent with earlier findings. For example, Wijsman et al. (6) reported that the transcriptome of bronchial brushes, as well as BALF cellularity and cytokine profiles, remained essentially unchanged six months after BT, including comparisons between treated and untreated airways. In our dataset, the expression of canonical T2-inflammatory response genes was similarly stable before and after procedure, suggesting relatively fixed immunologic endotypes defined by mRNA expression patterns. In contrast, one year after BT, we observed an increase in BALF eosinophils, suggesting rather enhanced asthmatic inflammation in more distal airway regions. This finding differs from previous reports describing reductions in BALF cytokines, including TGF-β and CCL5 after BT, while noting unchanged levels of proinflammatory cytokines, including IL-4, IL-5, IL-13, and IL-17A (6, 32–34). Additionally, we found no clear association between BALF eosinophilia and immune signature in BT-treated bronchi, suggesting that eosinophilic inflammation after BT may vary regionally within the bronchial tree. One plausible explanation may involve the compartmentalization of airway inflammation, with BAL reflecting rather distal airway regions beyond the primary reach of BT, potentially contributing to the observed divergence between airway and systemic measures. Interestingly, one potentially relevant finding was the trend toward an inverse relationship between BALF eosinophils and reduced oral corticosteroid use, suggesting that increased airway eosinophilia may be a response to steroid tapering following clinical improvement, although this requires further validation. In this context, our findings suggest that reducing chronic oral corticosteroid doses after BT may unmask localized eosinophilic inflammation in untreated or distal airway segments, thereby increasing airway eosinophilia (35). Evaluation of long-term clinical stability after BT requires larger cohorts and extended follow-up, particularly to enable comparisons across asthma subgroups with distinct immunological profiles.

Another aspect of our findings concerns the expression of remodeling−related genes in BT-treated airways and their relationship to structural airway parameters. BT produced only modest alterations in these transcripts, with *COL3A1* and *GDNF* showing the most apparent increase, and additional upward trends observed for *COL1A1*, *MMP2*, and *SNAI2*. Collagen−related genes in particular displayed coordinated upregulation and showed weak inverse association with ASM content. This pattern aligns with data published by Facciolongo et al. (18), who reported upregulation of fibrosis-associated genes, including *COL1A1* and *COL1A2*, two months after the first BT session. Our results extend these observations by demonstrating that a similar trend persists even one year after treatment. Moreover, a growing body of evidence suggests that baseline gene expression patterns, predominantly those associated with airway remodeling, may help predict BT responsiveness (18, 36). It is worth noting that the expression of remodeling markers was more strongly associated with the underlying immune pattern than with structural changes induced by BT treatment. For example, we observed a positive correlation between expression of collagen genes and the T3-signature, whereas a subset of genes, including *TGFB1* and *ZEB2*, correlated more strongly with the T2-signature. Nevertheless, subtle differences in how specific groups of profibrotic genes associated with structural measures were also apparent: expression of growth factors tended to correlate positively with RBM thickness and ASM content, while collagens showed the opposite trend. This observation aligns with our previous study on asthma (37), which also demonstrated an inverse relationship between submucosa collagen-I deposits and RBM thickness.

The effect of BT on lung function represents another important aspect of our results. Though basic spirometric parameters remained unchanged compared with baseline, we observed a marked reduction in the RV/TLC ratio, a marker of air trapping and lung hyperinflation. These findings are consistent with an observational study by Langton et al. (38), who demonstrated that BT leads to a significant reduction in airway resistance despite minimal changes in conventional spirometric indices. Similarly, Foo et al. (9) documented a reduction in ventilation heterogeneity based on functional lung MRI, a measure closely related to air trapping. Additional evidence from xenon−enhanced CT demonstrated improved air−trapping scores six weeks after BT (39). Collectively, these observations support the notion that improvements in air trapping may represent a key indicator of BT efficacy. Ongoing clinical trials are now evaluating advanced imaging techniques to better identify patients most likely to benefit from this intervention (40).

Our study has several limitations. First, the number of subjects studied was small, which prevented stratification into responders and non-responders. In addition, all analyses were performed on bronchial biopsies obtained from the BT-treated regions, without assessment of airway tissues beyond these areas. Because exact anatomical co−localization of repeat biopsies may not be fully ensured, some degree of sampling variability is unavoidable and may have affected histological morphometry results. Finally, as patients undergoing BT were selected based on increased L2 thickness, the study population was enriched for a remodeling−predominant phenotype, which limits the generalizability of our findings to the broader severe asthma population. Nevertheless, given the limited number of studies investigating bronchial gene expression before BT and after longer follow-up, we believe that our findings offer valuable insights into the mechanistic background of BT.

## Conclusions

Our data indicate that bronchial thermoplasty is a safe and effective treatment option for patients with severe refractory asthma that remains uncontrolled despite maximal optimized therapy. The procedure was associated with structural airway changes, including a reduction in ASM mass, and clinical benefits were likely related to a concomitant reduction in air trapping. These effects were accompanied by only modest increase in expression of a subset of profibrotic genes in treated bronchi, without significant alteration in immune-related gene expression, suggesting that the inflammatory endotypes of asthma remain relatively stable following BT therapy. In patients who experience improvement in asthma control after BT, cautious tapering of corticosteroids is advisable, as this may be associated with increased airway eosinophilia, potentially reflecting heterogeneous airway responses to the procedure. Further studies in larger asthma cohorts are needed to determine which asthma endotypes may benefit most from BT and to clarify how the procedure affects structural and molecular changes, particularly when comparing BT-treated proximal airway with untreated more distal regions.

## Data Availability

The original contributions presented in the study are included in the article/**Supplementary Material**. Further inquiries can be directed to the corresponding authors.
