# Supplementary material for: Effect of bronchial thermoplasty on airway inflammatory and profibrotic gene expression after one−year follow−up in severe asthma
Source: Front Immunol. 2026 Aug 7;17:1886894. doi: 10.3389/fimmu.2026.1886894 (PMC13493253; doi:10.3389/fimmu.2026.1886894)
Supplement: Supplementary file 1 [file SupplementaryFile1.docx]

**Supplementary materials**

**Effect of bronchial thermoplasty on airway inflammatory and profibrotic gene expression after one‑year follow‑up in severe asthma**

Bogdan Jakiela, Stanisława Bazan-Socha, Karolina Górka, Iwona Gross-Sondej, Sławomir Mikrut, Krzysztof Okoń, Piotr Sadowski, Anna Andrychiewicz, Tomasz Stachura, Marek Przybyszowski, Grażyna Bochenek, Krzysztof Sładek, and Jerzy Soja

**Corresponding authors**

Stanislawa Bazan-Socha, MD, PhD

Jerzy Soja, MD, PhD

Department of Internal Medicine, Faculty of Medicine, Jagiellonian University Medical College

Jakubowskiego 2

30-688 Krakow, Poland

Telephone: +48 12 4003054

Fax: +48 12 4003067

e-mail: stanislawa.bazan-socha@uj.edu.pl

e-mail: jerzy.soja@uj.edu.pl

**Supplementary Tables**

Supplementary Table S1. TaqMan gene expression assays (all from Applied Biosystems; Foster City, CA) used in the study.

| # | Gene symbol | Assay ID | Gene name |
| --- | --- | --- | --- |
| Inflammation panel | | | |
| 1 | *CCR3* | Hs99999027_s1 | C-C motif chemokine receptor 3 |
| 2 | *CLC* | Hs00171342_m1 | Charcot-Leyden crystal galectin |
| 3 | *CLCA1* | Hs00976287_m1 | chloride channel accessory 1 |
| 4 | *CPA3* | Hs00157019_m1 | carboxypeptidase A3 |
| 5 | *CST1* | Hs00606961_m1 | cystatin SN |
| 6 | *CXCL8* | Hs00174103_m1 | C-X-C motif chemokine ligand 8 |
| 7 | *DPP4* | Hs00897391_m1 | dipeptidyl peptidase 4 |
| 8 | *ELANE* | Hs00236952_m1 | elastase, neutrophil expressed |
| 9 | *GAPDH* | Hs99999905_m1 | glyceraldehyde-3-phosphate dehydrogenase |
| 10 | *GATA2* | Hs00231119_m1 | GATA binding protein 2 |
| 11 | *HDC* | Hs00157914_m1 | histidine decarboxylase |
| 12 | *IFNG* | Hs00989291_m1 | interferon gamma |
| 13 | *IL13* | Hs00174379_m1 | interleukin 13 |
| 14 | *IL17A* | Hs00174383_m1 | interleukin 17A |
| 15 | *IL1B* | Hs01555410_m1 | interleukin 1 beta |
| 16 | *IL33* | Hs00369211_m1 | interleukin 33 |
| 17 | *IL4* | Hs00174122_m1 | interleukin 4 |
| 18 | *IL6* | Hs00174131_m1 | interleukin 6 |
| 19 | *MPO* | Hs00165162_m1 | myeloperoxidase |
| 20 | *POSTN* | Hs01566750_m1 | periostin |
| 21 | *PRSS33* | Hs00541732_m1 | protease, serine 33 |
| 22 | *SERPINB2* | Hs01010736_m1 | serpin family B member 2 |
| 23 | *TNF* | Hs00174128_m1 | tumor necrosis factor |
| 24 | *TPSAB1*;*TPSB2* | Hs02576518_gH | tryptase beta 2 (gene/pseudogene);tryptase alpha/beta 1 |
| 25 | *TSLP* | Hs00263639_m1 | thymic stromal lymphopoietin |
| Remodeling panel | | | |
| 1 | *COL1A1* | Hs00164004_m1 | collagen type I alpha 1 |
| 2 | *COL3A1* | Hs00943809_m1 | collagen type III alpha 1 chain |
| 3 | *CTGF* | Hs00170014_m1 | connective tissue growth factor |
| 4 | *FGF2* | Hs00266645_m1 | fibroblast growth factor 2 |
| 5 | *FN1* | Hs01549976_m1 | fibronectin 1 |
| 6 | *GDF15* | Hs00171132_m1 | growth differentiation factor 15 |
| 7 | *GDNF* | Hs01931883_s1 | glial cell derived neurotrophic factor |
| 8 | *MMP2* | Hs01548727_m1 | matrix metallopeptidase 2 (gelatinase A, 72kDa gelatinase) |
| 9 | *MMP9* | Hs00957562_m1 | matrix metallopeptidase 9 (gelatinase B, 92kDa gelatinase) |
| 10 | *MUC5AC* | Hs01365616_m1 | mucin 5AC, oligomeric mucus/gel-forming |
| 11 | *MUC5B* | Hs00861595_m1 | mucin 5B, oligomeric mucus/gel-forming |
| 12 | *SMAD3* | Hs00969210_m1 | SMAD family member 3 |
| 13 | *SNAI2* | Hs00161904_m1 | snail family transcriptional repressor 2 |
| 14 | *TGFB1* | Hs00998133_m1 | transforming growth factor beta 1 |
| 15 | *TIMP1* | Hs01092512_g1 | TIMP metallopeptidase inhibitor 1 |
| 16 | *VEGFA* | Hs00900055_m1 | vascular endothelial growth factor A |
| 17 | *VIM* | Hs00958111_m1 | vimentin |
| 18 | *ZEB2* | Hs00207691_m1 | zinc finger E-box binding homeobox 2 |

Supplementary Table S2. Clinical characteristics of the study group.

|  | **BT**  (n=10) | **non-BT**  (n=20) | median difference or odds ratio  (95% CI) | p-value |
| --- | --- | --- | --- | --- |
| Age (years) | 50.5 [44-55] | 47 [40-56] | 3.5 (-6, 10) | 0.754 |
| Females (n, %) | 4 (40%) | 13 (65%) | 0.36 (0.09, 1.67) | 0.255 |
| BMI (kg/m^2^) | 25.4 [21.5-28.9] | 24.5 [22.3-28.7] | 0.9 (-4.5, 3.2) | 0.854 |
| Asthma duration (years) | 15.5 [4-31] | 16 [8.5-32.5] | -0.5 (-13, 8) | 0.610 |
| Positive SPT (n, %) | 6 (60%) | 10 (50%) | 1.5 (0.33, 5.8) | 0.709 |
| ACT (score) | 14 [11-19] | 12 [9-16] | 2 (-2, 6) | 0.353 |
| AQLQ (score) | 4.38 [3.22-5.71] | 3.75 [2.63-4.88] | 0.63 (-0.37, 1.88) | 0.207 |
| Exacerbations (n/year) | 5 [3-8] | 4 [3-8] | 1 (-2, 3) | 0.686 |
| ICS (µg/d) | 2200 [1950-2425] | 1450 [1000-1980] | 750 (125, 1200) | **0.015** |
| OCS (mg/d) | 10 [0-16] | 4 [0-15] | 6 (-4, 12) | 0.334 |
| OCS (n, %) | 7 (70%) | 12 (60%) | 1.56 (0.32, 6.74) | 0.702 |
| Blood eosinophils (cells/µL) | 156 [71.3-238] | 162 [97.3-432] | -6 (-253, 61) | 0.502 |
| Serum total IgE (IU/mL) | 67.5 [52.8-215] | 87.2 [17.8-254] | -19.7 (-113, 53) | 0.572 |
| Serum CRP (mg/L) | 1.5 [1-6.9] | 2.4 [1.3-4.7] | -0.9 (-2, 0.9) | 0.433 |
| ***Lung function*** |  |  |  |  |
| FVC (% predicted) | 78.4 [66.6-90.0] | 82.3 [72.5-97.8] | -3.9 (-19.5, 7) | 0.397 |
| FEV1 (% predicted) | 63.5 [47.0-71.3] | 62.7 [53.3-89.2] | 0.8 (-23, 10.2) | 0.468 |
| FEV1%FVC before BD | 62.6 [54.8-68.7] | 67.1 [52.8-74.5] | -4.5 (-13.4, 6.3) | 0.422 |
| FEV1%FVC after BD | 65.6 [60.0-75.3] | 71.3 [57.7-77.0] | -5.7 (-10.8, 6.8) | 0.713 |
| FAO (n,%) | 6 (60%) | 8 (40%) | 2.25 (0.49, 8.68) | 0.442 |
| FEV1.rev (% change after BD) | 8.0 [2.4-19.7] | 4.4 [-0.8-13.9] | 3.6 (-3.8, 10.8) | 0.281 |
| TLC (% predicted) | 107.5 [92.2-121.5] | 106.7 [96.3-120.6] | 0.8 (-14.3, 14.7) | 0.948 |
| RV (% predicted) | 153.5 [108.8-208] | 131.8 [121.3-174.9] | 21.7 (-28.6, 55) | 0.854 |
| RV/TLC (ratio) | 0.46 [0.41-0.51] | 0.42 [0.38-0.51] | -0.04 (-0.11, 0.06) | 0.481 |
| ***EBUS measurements*** |  |  |  |  |
| L1 (mm) | 0.185 [0.179-0.191] | 0.178 [0.170-0.183] | 0.007 (0.000, 0.014) | **0.032** |
| L2 (mm) | 0.208 [0.202-0.213] | 0.194 [0.184-0.201] | 0.014 (0.006, 0.023) | **0.001** |
| L3-5 (mm) | 0.979 [0.953-1.019] | 0.903 [0.865-0.940] | 0.076 (0.027, 0.130) | **0.004** |

Table 1 footnotes: Abbreviations: ACT, Asthma Control Test; AQLQ, Asthma Quality of Life Questionnaire; ASM, airway smooth muscle; BD, bronchodilator (short acting β2-agonist); BMI, body mass index; CRP, C-reactive protein; FAO, fixed airway obstruction (defined as FEV1%FVC post BD <70%); FEV1, forced expiratory volume in 1 second; FEV1.rev, reversibility of FEV1; FVC, forced vital capacity; ICS, inhaled corticosteroids (fluticasone-equivalent); OCS, oral corticosteroids (methylprednisolone-equivalent); RBM, reticular basement membrane; RV, residual volume; SPT, skin prick tests; TLC, total lung capacity.

Statistics: Data are presented as medians [Q1-Q3 quartiles]. Median differences with Mann-Whitney statistics (for continuous data) or odds ratios with contingency table statistics (for categorical data; Fisher’s exact test) are presented in separate columns. Significant differences (*p* < 0.05) are highlighted in bold.

Supplementary Figures

**Figure S1**

Figure S1 legend. **Endobronchial ultrasound (EBUS) assessment of bronchial wall layers and measurement workflow.** (**a**) EBUS is an advanced bronchoscopic ultrasonography technique that enables detailed visualization of the airway wall and adjacent anatomical structures. The procedure was performed using a 20 MHz radial miniprobe in conjunction with the EU‑ME1 processor system (Olympus, Tokyo, Japan). The schematic shows the setup, with the probe introduced through the working channel of the bronchoscope and a water‑filled balloon ensuring acoustic coupling to the bronchial wall. To ensure measurement consistency, EBUS was performed in each individual in four segmental bronchi of the right lower lobe: superior (RB6), anterior basal (RB8), lateral basal (RB9), and posterior basal (RB10). (**b**) Five images with clearly visible bronchial wall layers were selected for each location from video recordings obtained during bronchoscopy. The selected frames were saved as bitmap files and imported into feature extraction software for further analysis. Image processing included conversion from raster to vector format using a subpixel precision method. The borders of individual layers were manually indicated by a researcher blinded to patient data and sample status. The distance between selected points was measured and converted to millimeters. Multiple measurements were obtained for each image and averaged; then, the mean value across all locations was calculated and used for statistical analysis. Measurements were recorded to three decimal places (micrometer resolution). (**c**) Schematic illustration of bronchial wall layer segmentation as identified by EBUS, with measurement of L1, L2, and combined L3–L5 thickness. The image is a fragment of the photograph shown in ‘d’, rescaled and rotated. ASM, airway smooth muscle. (**d**) Representative EBUS-bronchoscopy image.

**Figure S2**

Figure S2 legend. **Associations between BT-induced changes in airway structure and remodeling gene expression**. (**a**) Heatmap showing Spearman correlation coefficients (rₛ) between changes in airway physiological and structural parameters (post–BT vs baseline) and changes in expression of remodeling-related genes. Genes are grouped into three indicated modules based on hierarchical clustering (Ward, Euclidean distance). The sidebar indicates the overall direction of parameter change at the one-year follow-up. *, *p* < 0.05. ASM, airway smooth muscle mass; BT, bronchial thermoplasty; ECM, extracellular matrix; FEV1, forced expiratory volume in 1 sec.; FEV1.rev, post-bronchodilator reversibility of FEV1; L2, endobronchial ultrasound layer reprsesenting most of submucosa; RBM, reticular basement membrane thickness; RV, residual volume; TLC, total lung capacity. (**b**) Representative scatter plots illustrating associations between changes in gene expression (fold change, log_10_; after-BT vs baseline), and airway structural parameters: change in L2 layer thickness (Δ-L2, mm) and change in RBM thickness (Δ-RBM, μm). Two representative genes are shown to highlight inverse or concordant associations; trends are depicted with fitted regression lines with 95% confidence intervals.
